# Acclimation temperature effects on locomotor traits in adult aquatic anurans (*X. tropicalis* and *X. laevis*) from different latitudes: possible implications for climate change

**DOI:** 10.1093/conphys/coz019

**Published:** 2019-05-20

**Authors:** Pablo Padilla, Valérie Ducret, Camille Bonneaud, Julien Courant, Anthony Herrel

**Affiliations:** 1 Département Adaptations du Vivant, UMR 7179, Centre national de la Recherche Scientifique/Muséum National d'Histoire Naturelle, 55 rue Buffon, Paris, France; 2 Centre for Ecology and Conservation, University of Exeter, Penryn, UK; 3 Evolutionary Morphology of Vertebrates, Ghent University, Ghent, Belgium

**Keywords:** Amphibians, locomotion, plasticity, temperature

## Abstract

Climate change is in part responsible for the 70% decline in amphibian species numbers worldwide. Although temperature is expected to impact whole-organism performance in ectotherms, reversible thermal acclimation has been suggested as a mechanism that may buffer responses to abrupt temperature changes. Here, we test for an effect of acclimation on locomotor performance traits (jump force and stamina) in adults of two predominantly aquatic and closely related frog species from different climatic regions, *Xenopus tropicalis* (tropical) and *Xenopus laevis* (temperate). We find significant effects of acclimation temperature on exertion capacity and for jump force in *X. tropicalis* but no effect of acclimation temperature on burst performance in *X. laevis*. Our results suggest that the two locomotor performance traits measured are differentially impacted by acclimation temperature in *X. tropicalis*. Our results further support the hypothesis that lower-latitude ectotherms might have greater acclimation capacity than high-latitude ones. Finally, our results highlight the importance of investigating multiple performance traits when evaluating how animals may cope with changes in temperature. Further work is required to evaluate the potential for acclimation in mitigating the negative impacts of climate change on amphibian populations.

## Teaser

Acclimation temperature affects locomotor performance in *X*. *tropicalis* but not in *X*. *laevis* at the adult stage. Expected increases in temperature due to global warming or deforestation are predicted to impact aquatic amphibians with limited opportunity for acclimation to mitigate these effects in temperate species.

## Introduction

The high thermal sensitivity of ectothermic organisms makes them particularly relevant for examining the consequences of climate change in terms of biodiversity erosion (van Berkum, 1986; Angilletta *et al*., 2002, Chown *et al*., 2010; Sinervo *et al*., 2010). For example, over 70% of amphibian species are currently experiencing worldwide declines, in part as a result of climate change (Alford and Richard, 1999; Blaustein and Kiesecker, 2002; Blaustein *et al*., 2010; Lawler *et al*., 2010, Wake and Vredenburg, 2008). Because amphibians have evolved temperature optima of many physiological traits that closely match their environmental temperatures (Navas *et al*., 2008; Angilletta *et al*., 2010), temperature changes will likely have physiological effects with possible downstream consequences given the fitness relevance of whole-organism physiology and performance (Herrel and Bonneaud, 2012a). Moreover, the immune system of amphibians is highly temperature dependent making them more susceptible to emerging infectious diseases as a result of changes in temperature (Raffel *et al*., 2006). Interestingly, despite the global decline in amphibians, some amphibians have become invasive pests and threaten local biodiversity (e.g. *Rhinella marina*; Shine, 2010*, Lithobates catesbeianus*; Ficetola *et al.*, 2010*, Xenopus laevis*; Measey *et al*., 2012; Courant *et al.*, 2018). How these species are capable of coping with changes in climatic conditions as they are moved from one region to another remains poorly understood but may provide insights into the mechanisms underlying temperature-related coping mechanisms.

Reversible thermal acclimation of a mature organism can be defined as a reversible shift in an organism's physiology resulting from a biological modification in response to a prolonged exposure to a change in temperature (Schlichting and Smith, 2002; Angilletta, 2009). Acclimation may consequently allow individuals to offset the direct impacts of temperature change, buffer animals to impacts from climate change and facilitate the invasion of new climatic zones. Thermal acclimation has been documented in many aquatic organisms, including fish and amphibians (Brattstrom, 1968; Hutchison *et al*., 1973; Feder *et al*., 1984; Feder, 1987; Marvin, 2003; Franklin *et al*., 2007; Wilson *et al*., 2007; Šamajová and Gvoždik, 2010; Madeira *et al*., 2016) and may therefore shape the responses of ectothermic organisms to environmental changes in temperature (Seebacher and Franklin, 2011; Winwood-Smith *et al*., 2015; Lachenicht *et al*., 2010; Vinagre *et al*., 2016). However, whereas fully aquatic anuran tadpoles readily acclimate to temperature changes (Wilson and Franklin, 1999; Wilson *et al*., 2000), in adult anurans the capacity to thermally acclimate their locomotor performance remains controversial (Wilson and Franklin, 2000). For example, Putnam and Bennett (1981) demonstrated that *Rana pipiens* could not thermally acclimate its locomotor performance at the adult stage. Rome (1983) provided further support for these findings by demonstrating a lack of thermal acclimation at a whole-muscle level. In contrast, Wilson *et al*. (2000) documented that adult *X. laevis* could acclimate their performance at 10°C. Miller and Zoghby (1986), in contrast, reported *R. pipiens* showing a thermal acclimation response at 12°C, yet no effect of thermal acclimation in *X. laevis* illustrating the lack of consensus between studies.

The ability of an ectotherm to thermally acclimate is thought to be linked to their opportunity for behavioural thermoregulation (Angilletta *et al.*, 2006). Since thermoregulation is usually more challenging in water than on land, thermal acclimation may also depend on whether animals are terrestrial or fully aquatic (Wilson *et al*., 2000). Indeed, showing a lower or even a complete lack of an acclimation response in terrestrial amphibians has been suggested to be due to the different thermal cues perceived in water and on land (Šamajová and Gvoždik, 2010). Recently, Seebacher *et al.* (2014) collated data from 202 ectotherms and suggested that thermal acclimation capacity of physiological traits was greater in ectotherms that live in more stable environments. They further suggested that aquatic animals might have a greater acclimation capacity compared to terrestrial ones. Consequently, tropical aquatic ectotherms living at lower latitudes can be expected to show a greater acclimation capacity. However, the opposite (i.e. ectotherms living at higher latitude having a greater acclimation capacity) was suggested previously by Janzen (1967). In fact, several studies have reported that tropical ectotherms (fish and arthropods, Vinagre *et al*., 2016; frogs, Feder, 1978, 1982; salamanders, Markle and Kozak, 2018; and lizards, Tsuji, 1988) showed lower or no acclimation capacity compared to their temperate relatives.

Here, we test whether the locomotor performance of adults in two closely related aquatic frog species from different latitudes, *Xenopus tropicalis* (tropical, low latitude) and *X. laevis* (temperate, high latitude), shows acclimation effects. Locomotor performance is temperature dependent (Herrel and Bonneaud, 2012a) and relevant to study in the context of predator escape, prey capture, reproduction (mate finding) and dispersal (Husak, 2006; James *et al.* 2007). However, different locomotor traits are important in different contexts with burst performance, for example, being likely selected for in the context of predator escape and prey capture (Vanhooydonck and Van Dame, 2003). In contrast, locomotor endurance or stamina is likely more important in the context of mate finding and dispersal. Trade-offs may exist in these locomotor traits due to their reliance of different underlying physiological traits (Herrel and Bonneaud, 2012b). As such, the thermal optima and the acclimation effects may also differ between these types of performance traits (Van Damme *et al*., 1991; Herrel and Bonneaud, 2012a). Finally, we also tested for sex differences in acclimation as males and females have previously been shown to differ in locomotor performance in both species (Herrel *et al*., 2012; Louppe *et al.*, 2017). Moreover, previous studies on other taxa have demonstrated sex-specific acclimation effects (Rogers *et al*., 2007). In summary, the objectives of this study were (i) to evaluate the capacity of adults of *X. tropicalis* and *X. laevis* to acclimate their burst locomotor performance (jumping) at low or high temperature; (ii) to explore the possible trade-offs between exertion and burst capacity in *X. tropicalis*; and (iii) to examine whether sexes differ in their thermal acclimation response.

## Materials and methods

### Animals

*Xenopus tropicalis* were caught in Cameroon in December 2009 and brought back to France (*N* = 102; 50 females, 52 males). Frogs were housed at the Station d’Ecologie Experimentale du CNRS at Moulis, where they were maintained in groups of 8 to 10 individuals in aquaria (60 × 30 × 30 cm). Animals were maintained at a temperature of 24°C, similar to the temperature of water bodies measured in the field (22–26°C; see Careau *et al*., 2014). *Xenopus laevis* were caught in a single pond in France (*N* = 32; 20 females and 12 males). Animals were brought back to the Function and Evolution (FUNEVOL) laboratory at the Muséum National d’Histoire Naturelle, Paris, France. Animals were maintained in groups of 6 to 10 individuals in 50-l aquaria in a *Xenopus* standalone racks with a recirculation system (Aquaneering). *Xenopus laevis* were maintained at a temperature of 23°C, close to their preferred body temperature and optimum temperature for burst performance (Casterlin and Reynolds, 1980; Miller, 1982). The frogs were given 1 month to recover and were then pit tagged (NONATEC) allowing the unique identification of each individual. Frogs were fed twice weekly with beef heart, earthworms or mosquito larvae. All experiments were approved by the Institutional ethics committee at the MNHN (#68-25). All applicable international, national and/or institutional guidelines for the care and use of animals were followed.

### Acclimation and performance trials

*Xenopus tropicalis* were split haphazardly in two groups. Group 1 was acclimated for 2 months at 29°C, close to the highest temperature they may encounter in the hottest month, and group 2 was maintained at 24°C, their laboratory temperature condition and close to the mean temperature of ponds at undisturbed forest sites. Next, performance was measured for all individuals at 24 and 29°C. Animals were then acclimated to the reciprocal temperatures for two months (group 1 at 24°C and group 2 at 29°C) and tested again at 24 and 29°C. Before the onset of performance measurements, animals were placed in individual containers with some water in an incubator, set to 24 or 29°C, for 1 hour. The same design was used for *X. laevis* but animals were tested and acclimated at 23°C based on the average water temperature measured in the ponds where they were caught during the active season. Performance measurements were repeated twice over the course of 1 day and animals were given at least 1 hour of rest between trials. While resting, frogs were returned to the incubator set at the test temperature. At the end of the performance trials, animals had their pit tag numbers recorded, and they were returned to their aquaria and given food. Animals were given at least 1 week of rest between the different performance measures (exertion versus burst performance). All animals were in good health at the end of the experiments.

Maximal exertion capacity was measured for *X. tropicalis* only and was measured by chasing each individual down a 3 m long circular track until exhaustion, indicated by the unwillingness to move when touched and the lack of a righting response (inability to turn when animals are place on their backs; see Herrel and Bonneaud, 2012a). We retained the time moved until exhaustion as our measure of endurance capacity. Maximal jump forces were measured for both species using a piezo-electric force platform (Kistler Squirrel force plate, 0.1 N). The force platform (20 × 10 cm) was connected to a charge amplifier (Kistler Charge Amplifier type, 9865), and forces were recorded at 500 Hz, transferred to the computer and recorded using BioWare software (Kistler). Animals were placed on the force plate, allowed to rest for a few seconds and then induced to jump by unexpectedly clapping our hands behind the frogs (Herrel *et al*., 2014). Two jump sessions with three to five jumps each on average were recorded, and the single most forceful jump was retained and used for further analyses. Forces in X, Y and Z-directions were extracted using the Kistler BioWare software, and the total resultant force (vector sum of the X, Y and Z-forces) was calculated.

**Figure 1:**
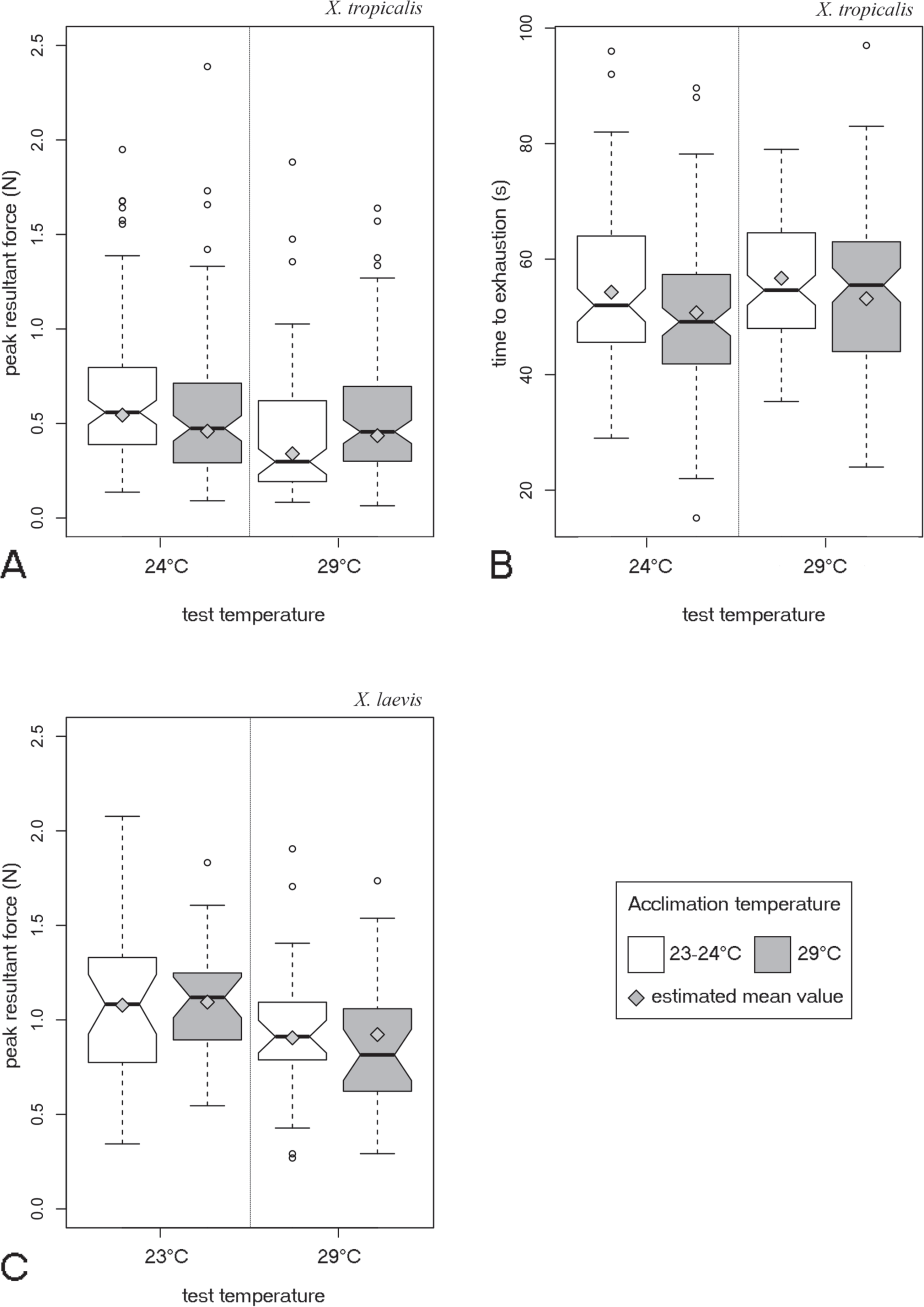
Effects of acclimation temperature and test temperature on the locomotor performance of two frog species. **(A)** Jumping performance measured as the peak resultant jump force in *X. tropicalis* (N_accl.24°C test24°C_: 101; N_accl. 29°C test 24°C_: 98; N_accl. 24°C test 29°C_: 99; N_accl. 29°C test 29°C_: 102). For jump force, the interaction between acclimation and test temperature was significant. **(B**) Exertion capacity measured as the time jumped until exhaustion in adult *X. tropicalis* (N_accl.24°C test24°C_: 102; N_accl. 29°C test 24°C_: 103; N_accl. 24°C test 29°C_: 102; N_accl. 29°C test 29°C_: 102), acclimation and test temperature had significant but independent effects. **(C**) Jumping performance in *X. laevis* (N_accl.24°C test24°C_: 31; N_accl. 29°C test 24°C_: 27; N_accl. 24°C test 29°C_: 31; N_accl. 29°C test 29°C_: 26) showing only an effect of test temperature. Notches illustrate the confidence intervals around the median. Values indicated by the grey diamonds are the estimated mean values from our mixed-effects model.

### Statistical analyses

For each model, we checked the linearity, normality and homoscedasticity of the residuals. Assumptions were mostly respected, except for normality and homoscedasticity in explaining the jump force of *X. tropicalis* and therefore this variable was log transformed. To control for pseudo-replication due to measurements of same individuals across test and acclimation treatment, we performed a mixed model effect analysis using the R package nlme (Bates *et al.*, 2015) with the individual identity as random factor on the intercept level. For each response variable (jump force and endurance capacity), we initially ran the model with acclimation temperature, test temperature, sex and all their interactions as main explanatory factor and body size (i.e. snout-vent length) as co-variable. We then proceed to remove each interaction factor, then single factors that were not significant in explaining the response variable (significance threshold was set at α = 0.05), and we compared goodness of fit between models using the anova() function in R. All statistical analyses were performed using the R software (R Development Core Team, 2018).

## Results

### *Xenopus tropicalis* exertion

In *X. tropicalis*, body size was not a good predictor for variation in endurance capacity (*t* = 0.69, *P* = 0.49) and individual identity explained part of the variance (standard deviations: intercept, 7.4; residual, 10.16). Exertion capacity was dependent on the acclimation temperature and the test temperature (acclimation: *X*^2^ = 11.39, *P* < 0.001; test: *X*^2^ = 5.09, *P* = 0.024). Exertion capacity was higher when individuals were measured at a higher test temperature (29°C) compared to a lower test temperature (24°C) (+2.3 seconds, *t* = 2.25, *P* = 0.025; Fig. 1B). However, individuals acclimated at a high temperature had a lower endurance capacity than individuals acclimated at a lower temperature (−3.4 seconds, *t* = 3.36, *P* < 0.001; Fig. 1B). We could not detect an interaction between acclimation temperature and test temperature (acclimation^*^test: *X*^2^ = 2.69, *P* = 0.10). However, we detected a tendency for an interaction between sex and acclimation (sex^*^acclimation: *X*^2^ = 3.74, *P* = 0.053) and between sex and temperature test (sex^*^test: *X*^2^ = 3.78, *P* = 0.052; Fig. 1C). An analysis of variance comparison between the final model (endurance ~ acclimation + test) and a model containing the latter two-way interactions (endurance ~ sex^*^acclimation + sex^*^test) indicated a lower AIC (respectively, 3175.99 versus 3173.42) and higher goodness of fit for the model containing the two-way interactions (L-ratio: 10.57, *P* = 0.032).

### *Xenopus tropicalis* burst performance

In *X. tropicalis*, body size predicted jump force (*t* = 5.44, *P* < 0.001) and individual identity explained part of the variance (standard deviations: intercept, 0.16; residuals, 0.60). *Xenopus tropicalis* had a higher jump force when measured at a test temperature of 24°C compared to a higher test temperature (test: *X*^2^ = 18.97, *P* < 0.001), but this effect was dependent on the acclimation treatment (acclimation^*^test: *X*^2^ = 12.69, *P* < 0.001). Individuals acclimated at 24°C had a 60% higher jump force when measured at the same test temperature compared to a high temperature (*t* = 5.56, *P* < 0.001; Fig. 1A), but when individuals were acclimated at a high temperature, their jump force was not dependent on the test temperature (*t* = 0.56, *P* = 0.58).

### *Xenopus laevis* burst performance

In *X. laevis*, body size predicted jump force (*t* = 2.11, *P* = 0.044) and individual identity explained part of the variance (standard deviations: intercept, 0.21; residuals, 0.29). Individuals measured at a test temperature of 23°C had a 19% higher jump force compared to individuals measured at a high temperature (*t* = 3.20, *P* = 0.002; Fig. 1D), this independently of their acclimation treatment (acclimation^*^test: *X*^2^ = 0.92, *P* = 0.34).

## Discussion

Our results support previous reports on ectotherms (Seebacher *et al.*, 2014) showing that species living at low latitude (tropical species, *X. tropicalis*) have greater acclimation capacity compared to species living at high latitude (temperate species, *X. laevis*), at least for jump performance*.* However, our results also highlight that those responses might not be the same across a range of traits, as in *X. tropicalis*, endurance capacity and jump force responded in different ways to acclimation temperature.

### Acclimation temperature effects are greater in *Xenopus* frogs from lower latitudes

The acclimation temperature effect on burst performance at high temperature in *X. tropicalis* is at odds with our results for *X. laevis*. *Xenopus tropicalis* is a tropical species of frog that rarely encounters extensive temperature variability in its environment (e.g. fluctuating between an average of 25.2 and 28.5°C between the hottest and the coldest month in southern Ivory Coast; Kouassi *et al*., 2010). In contrast, *X. laevis* encounters larger temperature fluctuations throughout the year in its native range (e.g. fluctuating between an average of 19.1 and 25.3°C in winter and summer in Cape Town, respectively**;**Levey, 1996), which may be even exacerbated in the invasive populations in France where ponds may freeze in winter. Our results on these two closely related species of different climatic regions tend to reject the hypothesis of Janzen (1967) (i.e. greater acclimation capacity at high latitude) and rather support the hypothesis that ectotherms living in more stable environments or at lower latitudes are more likely to exhibit thermal acclimation on their physiological traits (Temple and Johnston, 1998; Seebacher *et al*., 2014). Thus, acclimation likely does not provide the invasive *X. laevis* with a benefit enabling it to adapt to different climatic zones. More likely, this species possesses a broad thermal optimum allowing it to do well in a broad range of climatic zones. Yet, this remains to be tested. However, differences in acclimation capacity between our two species might also be explained by other differences besides latitude. For example, specimens of *X. laevis* were sampled from a single pond and consequently this population may have reduced genetic variability (and thus respond differently to acclimation treatment) compared to *X. tropicalis* sampled from different ponds. Rigorous studies combining ecological data of the local environment with complete information of each specimen (i.e. genetic diversity, relatedness between individuals and age) could resolve this issue and provide further insights into the observed species differences.

### Acclimation temperature differently affects two performance traits in *X. tropicalis*

Acclimation temperature affected stamina, independent of the test temperature. Indeed, irrespective of test temperature, animals were able to jump for longer when being acclimated at 24°C. However, burst performance capacity showed an interaction effect between acclimation temperature and test temperature. Indeed, animals acclimated at 24°C have their performance impacted by an acute change in test temperature. A previous study on *in vitro* muscle performance found that isolated limb muscles in *X. tropicalis* performed better (increase power output, shorter relaxation times) at higher temperature (up to 32°C; James *et al*., 2012). Whole-animal performance in this species, however, shows somewhat lower optimal temperatures for burst performance and endurance capacity (27 and 22°C; Herrel and Bonneaud 2012a). In the present study animals maintained similar burst performance capacity at both test temperatures when acclimated at 29°C. Thus, only acclimation temperatures near the optimal temperature allow animals to optimize burst performance regardless of test temperature.

### Interactions of sex and acclimation or test temperature in exertion capacity

In the frog *Litoria peronii*, males showed an acclimation effect in the performance of the calling muscles and the muscles used during amplexus (Rogers *et al*., 2007), whereas females did not. However, Rogers *et al.* (2007) detected no differences between sexes in the acclimation of the metabolic capacity of the ankle extensor muscle suggesting that only performance traits that are dimorphic may show interactions between sex and acclimation temperature. In our study, we only find a tendency for a sex-specific response to acclimation temperature, and to test temperature, for stamina in *X. tropicalis*, which may be due to the relatively small sample size. Females acclimated at 29°C seemed to have a compromised stamina capacity compared to any other group. Males measured at 29°C degree (test temperature) seemed to have enhanced performance. A comparison between our final model and a model containing the interaction between sex and acclimation, as well as between sex and test temperature, showed a higher goodness of fit for the model containing the two-way interactions suggesting a non-negligible effect of sex. Because sex-specific response to acclimation may have strong influence on the evolution of a given trait, future experiments with increased sample size for each sex are necessary to confirm those relationships.

### No acclimation response of burst performance to temperature in *X. laevis*

Acclimation temperature had no effect on burst performance in *X. laevis*, which is congruent with some other studies on burst performance in this species (Miller and Zoghby, 1986). However, prior studies showed contradictory results for acclimation at low temperature (10 or 12°C) with one study demonstrating an effect and the other not (Wilson *et al.,* 2000 versus Miller and Zoghby, 1986). As previous studies have demonstrated that early embryonic development can impact the ability of animals to acclimate at the tadpole stage (Seebacher and Grigaltchik, 2015), it would be of interest to test whether these effects are passed on to the adult stage. Similarly, it would be of interest to test whether exposure to different temperatures at the tadpole stage impacts the ability of adults to acclimate to different temperatures. If so, then this could explain contrasting results between these studies on thermal acclimation.

Overall, our results suggest that the tropical aquatic anuran *X. tropicalis* may have its locomotor performance compromised by temperature changes due to deforestation or increases in global thermal maxima. However, the capacity to mitigate these effects through acclimation may be dependent on the type of trait measured. In the context of global climate change and habitat fragmentation, temperatures in the ponds of these animals in the wild are expected to increase (Beaumont *et al*., 2011). Our results suggest that if *X. tropicalis* faces long-term conditions of high temperature, they may suffer a decrease in stamina regardless of any daily temperature changes. Thermal acclimation at high temperature of burst performance capacity seems to not be affected by daily temperature changes. However, when acclimating this trait at low temperature, an acute increase of daily temperature seems to negatively affect performance resulting in potential consequences for their ability to escape predators or capture prey. On the other hand, the temperate aquatic anuran *X. laevis* appears to not acclimate its burst performance and is thus directly impacted by changes in daily temperature, with jump force being negatively impacted by higher temperatures. Quantifying the thermal dependence of performance in this worldwide invasive species may consequently provide more accurate predictions of their future distribution limits under different global warming scenarios.

## Supplementary Material

Padilla_Supplementary_Table_coz019
