# Supplementary material for: Acclimation temperature effects on locomotor traits in adult aquatic anurans (X. tropicalis and X. laevis) from different latitudes: possible implications for climate change
Source: Conserv Physiol. 2019 May 20;7(1):coz019. doi: 10.1093/conphys/coz019 (PMC6528024; doi:10.1093/conphys/coz019)
Supplement: Padilla_Supplementary_Table_coz019 [file padilla_supplementary_table_coz019.docx]

**Supplementary Table 1**: summary of the effects of acclimation, sex, and test temperature on performance for each species. Data are mean values ± standard deviations. M, male; F, female.

| species | test temperature | acclimation temperature | sex | time to exhaustion (s) | peak force (N) |
| --- | --- | --- | --- | --- | --- |
| *Xenopus tropicalis* | 24 °C | 24°C | M | 55.19 ± 13.52 | 0.55 ± 0.37 |
|  |  |  | F | 55.13 ± 15.18 | 0.74 ± 0.40 |
|  |  | 29°C | M | 51.04 ± 12.7 | 0.55 ± 0.44 |
|  |  |  | F | 48.72 ± 13.22 | 0.59 ± 0.34 |
|  | 29°C | 24°C | M | 56.72 ± 10.2 | 0.36 ± 0.27 |
|  |  |  | F | 54.93 ± 10.87 | 0.51 ± 0.37 |
|  |  | 29°C | M | 57.99 ± 12.99 | 0.52 ± 0.40 |
|  |  |  | F | 50.22 ± 11.3 | 0.53 ± 0.25 |
| *Xenopus*  *laevis* | 23°C | 23°C | M | not measured | 0.99 ± 0.51 |
|  |  |  | F |  | 1.10 ± 0.40 |
|  |  | 29°C | M | not measured | 1.00 ± 0.21 |
|  |  |  | F |  | 1.15 ± 0.32 |
|  | 29°C | 23°C | M | not measured | 0.87 ± 0.34 |
|  |  |  | F |  | 0.97 ± 0.40 |
|  |  | 29°C | M | not measured | 0.90 ± 0.34 |
|  |  |  | F |  | 0.87 ± 0.37 |
